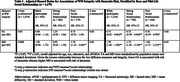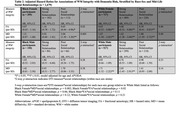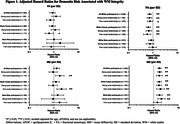# The Modifying Effects of Sex, Race, and Social Relationships on the Association between White Matter Integrity and Dementia Risk: The Atherosclerosis Risk in Communities (ARIC) Neurocognitive Study

**DOI:** 10.1002/alz.093711

**Published:** 2025-01-09

**Authors:** Surabhee Eswaran, Rebecca F. Gottesman, David S. Knopman, Silvia Koton, Anna M. Kucharska‐Newton, Albert C. Liu, Chelsea Liu, Pamela L. Lutsey, Thomas H. Mosley, Priya Palta, A. Richey Sharrett, Kevin J. Sullivan, Keenan A. Walker, Renee Groechel

**Affiliations:** ^1^ Tulane University, New Orleans, LA USA; ^2^ National Institute of Neurological Disorders & Stroke Intramural Research Program, National Institute of Health, Bethesda, MD USA; ^3^ Mayo Clinic, Rochester, MN USA; ^4^ The Stanley Steyer School of Health Professions, Tel Aviv Univeristy, Tel Aviv Israel; ^5^ University of North Carolina Gillings School of Global Public Health, Chapel Hill, NC USA; ^6^ George Washington University‐Milken Institute of Public Health, Washington, DC USA; ^7^ University of Minnesota School of Public Health, Minneapolis, MN USA; ^8^ University of Mississippi Medical Center, Jackson, MS USA; ^9^ University of North Carolina Chapel Hill, Chapel Hill, NC USA; ^10^ Johns Hopkins University Bloomberg School of Public Health, Baltimore, MD USA; ^11^ University of Mississippi Medical Center, The MIND Center, Jackson, MS USA; ^12^ Laboratory of Behavioral Neuroscience, National Institute on Aging, Intramural Research Program, Baltimore, MD USA; ^13^ National Institute of Neurological Disorders and Stroke, Intramural Research Program, Bethesda, MD USA

## Abstract

**Background:**

Weakened white matter (WM) integrity is highly associated with dementia risk. Still, not everyone with WM changes develops dementia, suggesting the important role modifiable lifestyle factors may have in reducing dementia risk. We investigated how social relationships in mid‐life may modify the association between WM integrity and incident dementia risk within race and sex subgroups.

**Method:**

In 1,679 Atherosclerosis Risk in Community (ARIC) study participants who were dementia‐free in mid‐life, psychosocial health was assessed via self‐reported questionnaires and participants were classified as having strong or poor social relationships in mid‐life (visit 2: 1990‐1992). Through diffusion tensor imaging, WM integrity was evaluated with 3T brain MRI (visit 5: 2011 – 2013); fractional anisotropy (FA) and mean diffusivity (MD) were estimated. Incident dementia cases were identified from visit 5 through December 31, 2019, with ongoing surveillance. Relative contributions of social relationships and WM integrity to incident dementia were evaluated in Cox‐proportional hazard regression models with race and race‐sex as interaction terms, and in stratified models (White Females, Black Females, White Males, Black Males).

**Result:**

Significant three‐way interactions were observed between race, WM integrity, and mid‐life social relationships (p‐interaction<0.01). In Black participants with strong mid‐life social relationships, higher MD (hazard ratio (HR): 0.93, 95% CI: 0.63 – 1.38) and lower FA (HR: 1.25, 95% CI: 0.84 – 1.86) were not associated with the risk of dementia, although in Black participants with poor social relationships, higher MD remained a significant risk factor for dementia. However, across all White participants, higher MD and lower FA were associated with the risk of dementia, regardless of mid‐life social relationships (Table 1; Figure 1). Analysis of three‐way interactions between race‐sex, WM integrity, and mid‐life social relationships suggested that effect modification by mid‐life social relationships was most evident in Black Females, respective to other groups (Table 2; Figure 1).

**Conclusion:**

The contribution of WM integrity to dementia risk may be lower in Black participants with strong social relationships in mid‐life, particularly in Black Females. Further understanding of how contextual social factors may influence brain health in diverse populations should be examined in future studies and intervention efforts.